# *Staphylococcus aureus* Internalized by Skin Keratinocytes Evade Antibiotic Killing

**DOI:** 10.3389/fmicb.2019.02242

**Published:** 2019-09-24

**Authors:** Arwa Al Kindi, Abdullah M. Alkahtani, Mayimuna Nalubega, Cecile El-Chami, Catherine O’Neill, Peter D. Arkwright, Joanne L. Pennock

**Affiliations:** ^1^Lydia Becker Institute of Immunology and Inflammation, The University of Manchester, Manchester, United Kingdom; ^2^Division of Infection, Immunity and Respiratory Medicine, The University of Manchester, Manchester, United Kingdom; ^3^College of Medicine, King Khalid University, Abha, Saudi Arabia; ^4^Division of Musculoskeletal and Dermatological Sciences, The University of Manchester, Manchester, United Kingdom

**Keywords:** *Staphylococcus aureus*, skin, keratinocyte, internalization, antibiotic sensitivity, rifampicin

## Abstract

*Staphylococcus aureus* causes the majority of skin and soft tissue infections. Half of patients treated for primary skin infections suffer recurrences within 6 months despite appropriate antibiotic sensitivities and infection control measures. We investigated whether *S. aureus* internalized by human skin keratinocytes are effectively eradicated by standard anti-staphylococcal antibiotics. *S. aureus*, but not *S. epidermidis*, were internalized and survive within keratinocytes without inducing cytotoxicity or releasing the IL-33 danger signal. Except for rifampicin, anti-staphylococcal antibiotics in regular clinical use, including flucloxacillin, teicoplanin, clindamycin, and linezolid, did not kill internalized *S. aureus*, even at 20-fold their standard minimal inhibitory concentration. We conclude that internalization of *S. aureus* by human skin keratinocytes allows the bacteria to evade killing by most anti-staphylococcal antibiotics. Antimicrobial strategies, including antibiotic combinations better able to penetrate into mammalian cells are required if intracellular *S. aureus* are to be effectively eradicated and recurrent infections prevented.

## Introduction

*Staphylococcus aureus* (*S. aureus*) colonizes the skin in 20–30% of the population and causes 80–90% of all skin and soft tissue infections in humans worldwide (Tong et al., 2015; Dayan et al., 2016; Peterson and Schora, 2016). In the United States, the incidence of hospitalization for *S. aureus* infections more than doubled from 57/100,000 population in 2001 to 117/100,000 population in 2009 (Suaya et al., 2014). A large US-based, multicenter, retrospective cohort of 50 million insured individuals found that between 2005 and 2010, there were 2.3 million ambulatory and hospital encounters of patients 0–65 years old with skin and soft tissue infections (Miller et al., 2015b; Kaye et al., 2019). Infections are more troublesome in patients with disrupted epidermal stratum corneum, a common occurrence in atopic dermatitis where colonization reaches almost 100% (Kobayashi et al., 2015; Tauber et al., 2016; Meylan et al., 2017).

Recurrent *S. aureus* skin infections are reported in 39% of patients within 3 months, and >50% within 6 months of initial infection, necessitating repeated courses of antibiotics and increasing the risk of antibiotic resistance (Miller et al., 2015a; Geoghegan et al., 2018). The reasons for reinfection are multi-factorial and an important cause is cross- and re-infection from family members, healthcare providers, pets, and fomites (Montgomery et al., 2015). MRSA is also a major challenge, with their rapid spread in both the community and hospital settings since initial identification in 1961. MRSA colonization rates vary between 0.2 and 7.4% in the community, with subsequent spread to two-thirds of household contacts. Hospital patients have a much higher prevalence of MRSA, of between 20 and 40% (Bassetti et al., 2017; Turner et al., 2019).

The main question we set out to address in this study is why some patients develop chronic or recurrent infections with the same *S. aureus* clone, even after apparently adequate courses of antibiotics for proven methicillin-sensitive strains and appropriate measures to combat cross-contamination (Byrd et al., 2017; O’Gara, 2017). We hypothesized that internalization of *S. aureus* by keratinocytes allows bacteria to evade both normal host immunity and anti-staphylococcal antibiotics. Evidence from animal and human studies have previously demonstrated that *S. aureus* are not only phagocytosed by neutrophils and macrophages, but can also be internalized by epidermal keratinocytes (Mempel et al., 2002; Kintarak et al., 2004; Bur et al., 2013). However, these non-professional phagocytes lack the cytoplasmic organelles needed to kill *S. aureus* (Kubica et al., 2008). To test our hypothesis, we use a number of imaging techniques to demonstrate that *S. aureus*, but not *S. epidermidis* (SE), are not only internalized by primary normal human epidermal keratinocytes (NHEK) but do not induce cytotoxicity or an inflammatory response. Importantly, we have demonstrated that most standard anti-staphylococcal antibiotics, which are able to kill methicillin-sensitive *S. aureus* in culture growth media are not able to kill the microbes once internalized by keratinocytes.

## Materials and Methods

### Materials

**Table:** 

**Reagent or resource**	**Source**	**Identifier**
**Bacterial strains**		
Clin1-SA	Professor A. McBain, University of Manchester, United Kingdom	–
Clin2-SA-NC2669	Public Health England	NCTC 2669
Lab1-SA-SH1000 and its isogenic fnbA fnbB	Professor J. Geoghegan, University of Dublin, Ireland	8325-4
Lab2-SA-GFP	Professor A. Horswill, University of Colorado, United States	AH2547
*S. epidermidis*	Dr. G. Xia, University of Manchester, United Kingdom	–
**NHEK culture**		
Primary Normal Human Epidermal Keratinocytes (NHEK)	PromoCell, Heidelberg, Germany	C-12002
Clindamycin	Sigma–Aldrich, United Kingdom	C2250000
Flucloxacillin	Wockhardt Ltd., United Kingdom	10427812
Teicoplanin	Sigma–Aldrich, United Kingdom	Y0001102
Penicillin (100 U/ml) and streptomycin (0.1 mg/ml)	Sigma–Aldrich, United Kingdom	P4333
**Antibiotics**		
Etest-RIFAMPICIN	bioMérieux Ltd., Basingstoke, United Kingdom	412450
Etest-LINEZOLID	bioMérieux Ltd., Basingstoke, United Kingdom	412396
Etest-CLINDAMYCIN	bioMérieux Ltd., Basingstoke, United Kingdom	412315
Etest-OXACILLIN	bioMérieux Ltd., Basingstoke, United Kingdom	412432
Etest-TEICOPLANIN	bioMérieux Ltd., Basingstoke, United Kingdom	412461
Rifampicin	Sigma–Aldrich, United Kingdom	R3501
Linezolid	Sigma–Aldrich, United Kingdom	PZ0014
**Antibodies and fluorescent labeling**		
FITC isomer	Sigma–Aldrich, United Kingdom	F7250
Alexa Fluor^®^ 647 Mouse Anti-Human Cytokeratin 14/15/16/19 (clone KA4)	BD Bioscience, United Kingdom	563648
4′,6-Diamidino-2-phenylindole (DAPI)	New England Biolabs, Canada	4083S
Claudin-I (Rabbit, polyclonal, MH25)	Thermo Fisher Scientific, United Kingdom	71-7800
Texas red goat antirabbit antibody	Life Technologies, United States	T-2767
**Software**		
FlowJo software (V10)	Flow Jo, Tree Star	https://www.flowjo.com/solutions/flowjo/
IDEAS 6.2	Amnis support	https://amnis.com/
Imaris X64 9.2.1 (Bitplane, Oxford, United Kingdom)	Bitplane	https://imaris.oxinst.com/
ImageJ	NIH Image	https://imagej.net/ImageJ

### *S. aureus* and *S. epidermidis* Species and Strains

*Staphylococcus aureus* (Clin-SA) was isolated from a chronic skin wound (courtesy of Professor A. McBain, University of Manchester, United Kingdom). Lab strain of *S. aureus* SH1000 (Lab1-SA-SH1000) and its isogenic *fnbA fnbB* mutant were kindly provided by Professor J. Geoghegan, University of Dublin, Ireland. *S. aureus* NCTC 2669 was purchased from Public Health England. *S. aureus*-Lab2-SA-GFP (Green Fluorescent Protein), chloramphenicol-resistant strain was provided by Professor A. Horswill, University of Colorado, United States. SE was a gift from Dr. G. Xia, University of Manchester, Manchester, United Kingdom.

### Preparation and Fluorescent Labeling of *S. aureus* and *S. epidermidis*

Bacterial count was assessed by spectrophotometry (600 nm) and the Miles and Misra method. For FITC labeling, *S. aureus* and SE were grown on nutrient agar and incubated at 37°C for 18 h. One colony was inoculated into 13 ml nutrient broth and incubated at 37°C overnight to achieve 10^10^ CFU/ml. Ten milliliters of the overnight culture was washed in phosphate buffered saline (PBS), centrifuged (1,600 × *g*, 5 min), and resuspended in 10 ml of 0.1 M carbonate buffer (pH 9) containing 100 mg/l FITC isomer (Sigma–Aldrich, United Kingdom) for 1 h at room temperature, according to manufacturer instructions. Bacterial cultures were centrifuged at 600 × *g* for 5 min, washed with PBS, resuspended in 1% glycerol, and stored at −80°C until needed.

### Primary Normal Human Epidermal Keratinocytes Culture

Normal Human Epidermal Keratinocytes (PromoCell, Heidelberg, Germany) were grown to 80% confluence before passage in keratinocyte complete growth media (with supplements) (PromoCell, Heidelberg, Germany) at 37°C, 5% CO_2_. Cells were detached using TrypLE (Thermo Fisher Scientific, United Kingdom) according to manufacturer instructions. Primary cells were used between passage one and four before disposal.

### Assessment of Bacterial Internalization

#### Cell Culture

Normal Human Epidermal Keratinocytes were seeded in 24-well plates (1 × 10^6^ cells/ml) and incubated with 10^7^ CFU/ml of either FITC- or GFP-labeled bacteria in complete keratinocyte growth media for 1 h (37°C, 5% CO_2_). Two percent penicillin/streptomycin (2% P/S) were then added to each well and left for 30 min to kill extracellular bacteria. Cells were analyzed between 1 and 24 h post-internalization.

For both flow cytometry and Amnis^®^ experiments, NHEK were washed with PBS and detached using 0.025% trypsin/0.01% EDTA (PromoCell, Heidelberg, Germany) for 5 min at 37°C, 5% CO_2_ followed by trypsin neutralization (0.05% trypsin inhibitor from soybean and 0.1% bovine serum albumin).

#### Flow Cytometry

Initially, internalization of bacteria by NHEK was studied by standard flow cytometry (BD FACS Canto II, BD Biosciences, United Kingdom) and analyzed using FlowJo software (Tree Star V10). Cells were prepared as described above. NHEK inherent autofluorescence was excluded using a (PerCP)-Cy5^+^/FITC^+^ gate. Single FITC^+^ cells representing green florescent protein (GFP)-*S. aureus* positive NHEK were taken forward for analysis.

Annexin V and DAPI were used to assess cell death. Cells were incubated with Annexin V-APC (eBioscience, United Kingdom) in cell staining buffer (Biolegend, United Kingdom) for 20 min. Cells were then washed and DAPI (New England Biolabs, United Kingdom) added to a final concentration of 0.25 μg/ml prior to flow cytometry analysis.

Internalization was also assessed using Amnis^®^ (ImageStream^®^ Mark II Imaging Flow Cytometer, Merck, United Kingdom), which allows microscopic visualization of flow cytometry gated cells. NHEK were prepared as described above. After P/S treatment, cells were incubated in complete keratinocyte growth media for 4 h (37°C, 5% CO_2_) then fixed with 4% paraformaldehyde (10 min, RT) and permeabilized (0.1% Triton-X, 10 min). NHEK were resuspended with 1 μg/ml of Alexa Fluor^®^ 647 mouse anti-human cytokeratin 14/15/16/19 (clone KA4, BD Bioscience, United Kingdom) in permeabilization buffer to identify primary keratinocytes (20 min, RT in the dark), washed, and resuspended. As for flow cytometry analysis, NHEK inherent autofluorescence was excluded using a (PerCP)-Cy5^+^/FITC^+^ gate. Cytokeratin 14/15/16/19^+^/*S. aureus-*FITC^+^ cells were gated and sorted for Amnis^®^ analysis using IDEAS 6.2.

#### Confocal Microscopy

Confocal microscopy was also performed to confirm internalization of Lab2-SA-GFP by NHEK. 1 × 10^6^cells/ml were incubated with 10^7^ CFU/ml Lab2-SA-GFP (1 h, 37°C, 5% CO_2_) then washed, treated with 2% P/S (30 min), and incubated in media for 4 h (37°C, 5% CO_2_). NHEK were fixed with 4% PFA and permeabilized using 0.1% Triton-X. Cells were stained with CellMask Deep Red stain according to manufacturer’s instructions (Thermo Fisher Scientific, United Kingdom). Images were collected on a Leica TCS SP5 AOBS inverted confocal using a 100×/1.40 immersion oil objective and 4× confocal zoom. The confocal settings were as follows: pinhole 1 airy unit, scan speed 400 Hz bidirectional, format 512 × 512. Images were collected using HyD detectors with the following detection mirror settings; FITC 493-589 nm, Texas red 599-615 nm using the 488 (10) and 594 nm (1%) laser lines, respectively. When it was not possible to eliminate cross-talk between channels, the images were collected sequentially. When acquiring 3D optical stacks, the confocal software was used to determine the optimal number of Z sections. Only the maximum intensity projections of these 3D stacks are shown in the section “Results.” Imaris X64 9.2.1 (Bitplane, United Kingdom) image analysis software was used to analyze the data.

### Inhibition of Internalization

To assess inhibition of *S. aureus* internalization, cells were prepared as described above. Media in each well was discarded and cells washed with PBS. Where detailed in the text, NHEK were pre-treated with anti-α5β1 integrin antibody (clone JBS5, Sigma–Aldrich, United Kingdom) for 30 min (37°C, 5% CO_2_) in complete keratinocyte media. Pre-treated NHEK were then infected with *S. aureus* (10^7^ CFU/ml) in the presence of inhibitor for 1 h. Media containing *S. aureus* and inhibitors was discarded and cells were treated with 2% P/S for 30 min. Cells were then washed with PBS and detached for flow cytometry analysis as described above.

### Antibiotic MIC Determination

The bactericidal properties of flucloxacillin (Wockhardt Ltd., United Kingdom), clindamycin, teicoplanin, linezolid, and rifampicin (all Sigma–Aldrich, United Kingdom) were assessed. Minimum inhibitory concentration (MIC) for each antibiotic was determined by both Etest reagent strips (bioMérieux Ltd., Basingstoke, United Kingdom) and the Microtitre Broth Dilution Method as described previously (Wiegand et al., 2008). Specific MIC for each antibiotic is detailed in the text.

### *In vitro* Infection of NHEK With *S. aureus* and Antibiotics Assay

Normal Human Epidermal Keratinocyte cells were seeded in 24-well plates (5 × 10^4^ cells/ml). When confluent, cells were infected with 10^7^ CFU/ml *S. aureus* (diluted in keratinocyte growth media from overnight culture) for 1 h at 37°C followed by treatment with 2% P/S for 1 h to eliminate extracellular *S. aureus*. Cells were washed with PBS and incubated in complete keratinocyte media for 4 h (37°C, 5% CO_2_). Media was then replaced with or without anti-staphylococcal antibiotics in complete media for a further 24 h, as outlined in the text. Fifty microliters of supernatant was then removed for culture on nutrient agar plates in order to quantify extracellular *S. aureus*. Cells were then washed three times with PBS, treated with 2% P/S for 1 h, and lysed in 300 μl of PBS using mini-scrapers (VWR International, United Kingdom) and vigorous vortex. Cell lysates were cultured on nutrient agar using serial dilution to assess the number of intracellular *S. aureus*. For quantification of CFU, three technical replicates were performed for each well.

### *Ex vivo* Human Skin Organ Culture and Infection With GFP-*S. aureus*

Human skin was obtained following liposculpture procedures performed on healthy adult patients. The study was approved by the North West Research Ethics committee (REC ref. 14/NW/0185) and all of the patients gave written informed consent. A 4-mm diameter biopsy punch (Integra^TM^ Miltex^TM^, Fisher Scientific) was placed in a Thincert cell culture insert with a pore size of 0.4 μm (Greiner Bio-One, United Kingdom) and cultured in a six-well plate, creating an air–liquid interface. The culture medium consisted of William’s E media (Thermo Fisher) supplemented with 1% (v/v) L-glutamine, 0.02% (v/v) hydrocortisone, and 0.1% (v/v) insulin. The surface of the biopsy was loaded with 3 μl of 10^7^ CFU/ml Lab2-SA-GFP and incubated at 37°C, 5% CO_2_ for 3 h. After culture the biopsies were snap-frozen in liquid nitrogen and stored at −80°C until use.

### Immunofluorescence Staining

Skin tissue blocks were cut to 5 μm depth (−20°C, OFT Cryostat; Bright Instruments, United Kingdom) in triplicate onto Superfrost^TM^ Ultra Plus Adhesion slides (Thermo Fisher Scientific, United Kingdom). Sections were labeled and stored at −20°C until needed.

Frozen sections were air dried (10 min, RT), then fixed with methanol:acetone (50:50 v/v) for 20 min at −20°C. Slides were washed in tris-buffered saline (TBS) three times (5 min each) then permeabilized with 0.1% Triton-X100 (5 min, RT). Ten percent normal goat serum (NGS, Vector Laboratories Inc., United States) was used as diluent and to block non-specific binding (1 h, RT). Sections were stained with Claudin-I (rabbit anti-human polyclonal, MH25, Thermo Fisher Scientific, United Kingdom) diluted 1:50 in diluent and incubated overnight at 4°C. Sections were washed in TBS-0.05% Tween-20 and incubated with Texas^TM^ red goat anti-rabbit antibody (Life Technologies Corporation, Thermo Fisher) diluted 1:100 in block and incubated in the dark for 1 h at RT before washing again. Finally, sections were mounted with VETASHIELD^®^ HardSet^TM^ mounting medium (Vector Laboratories Inc., United States).

### Statistical Analysis

Statistical comparisons were made using one-way ANOVA, with Dunnet’s *post hoc* test using GraphPad Prism. *P* < 0.05 was considered statistically significant.

## Results

### *S. aureus* but Not *S. epidermidis* Are Internalized by Human Skin Keratinocytes (NHEK)

Previous work has shown that *S. aureus* can be internalized by immortalized human HaCaT keratinocytes (Mempel et al., 2002; Bur et al., 2013). Here we extend these observations using both FITC labeled and GFP expressing bacteria.

**FIGURE 1:**
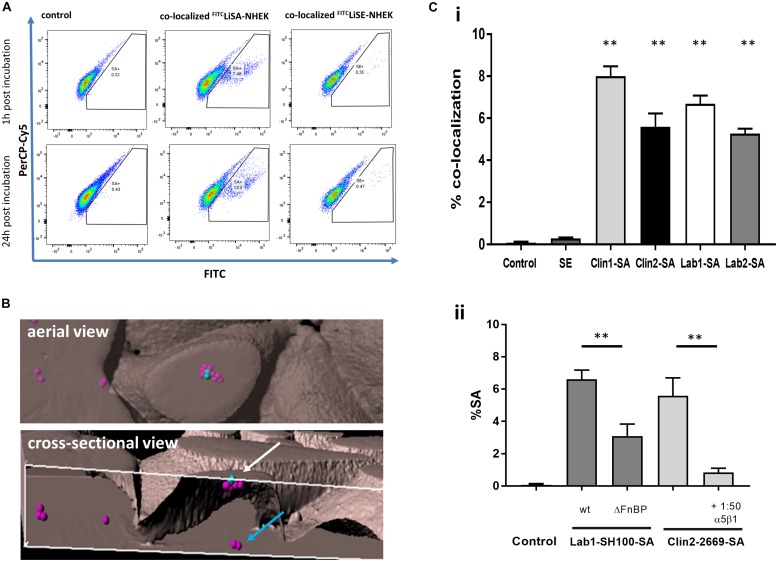
Internalization of live *S. aureus* but not live SE into primary Normal Human Epidermal Keratinocytes (NHEK) via α5β1-integrin. **(A)** NHEK were co-cultured with FITC-labeled live *S. aureus* (^FITC^LiSA) or SE (^FITC^LiSE) at 37°C for 1 h, followed by washing with penicillin/streptomycin to kill extracellular bacteria. Culture was continued for 1 or 24 h and co-localization determined by flow cytometry. Autofluorescence is shown on PerCp-Cy5/FITC double positive population. Controls represent NHEK without bacterial co-culture. Flow cytometry scatter plots and images are representative of three independent experiments with technical triplicates. **(B)** Internalization of ^GFP^*S. aureus* by NHEK was confirmed by 3D confocal microscopy of NHEK after co-culture with 10^7^ CFU/ml ^GFP^*S. aureus* for 1 h at 37°C. Representative aerial and cross-sectional views of extracellular (blue spots) and intracellular (pink spots) *S. aureus*. **(Ci)** FITC-labeled clinical (Clin1-SA and Clin2-SA-NCTC2669) and laboratory (Lab1-SA-SH1000 and Lab2-SA-GFP) isolates of *S. aureus* were internalized to a similar extent by NHEK. **(Cii)** Fibronectin binding protein *S. aureus* mutants were less effectively internalized than the wild-type Lab1-SA-SH1000. Internalization of FITC-labeled Clin2-SA-NCTC2669 was inhibited with blocking anti-α5β1-integrin antibody. All experiments were performed in triplicate. Statistical comparison in comparison to WT was performed by one-way ANOVA. ^∗∗^*p* < 0.0001.

Firstly, by flow cytometry we demonstrated that FITC-*S. aureus* but not FITC-SE co-localize with primary NHEK. In these experiments, NHEK were incubated with 10^7^ CFU bacteria for 1 h before incubating with P/S to kill extracellular bacteria. Incubation was continued for either 1 or 24 h (Figure 1A). Staining was not due to inherent autofluorescence as shown by PerCP-Cy5/FITC linear co-localization. Co-localization of *S. aureus* with NHEK was significantly higher (7.5 ± 2.2%, *p* < 0.01) than control wells. There was no significant co-localization of SE with NHEK.

**FIGURE 2:**
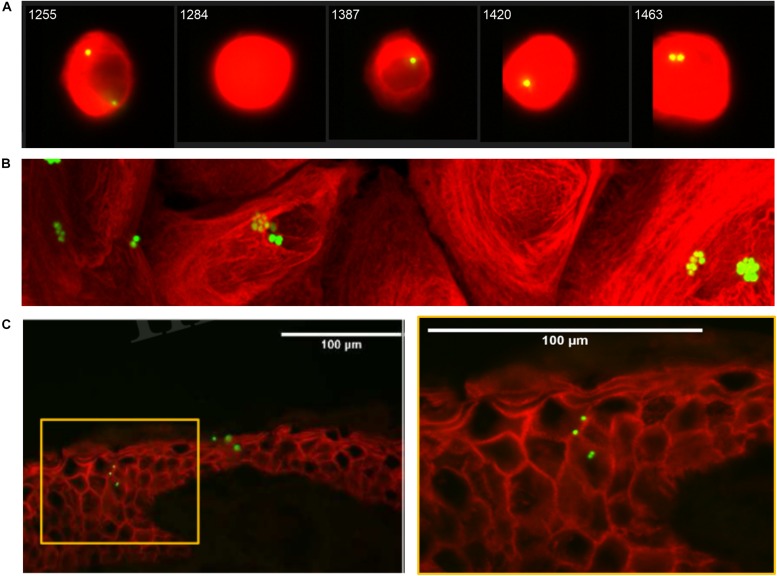
Internalization of ^GFP^*S. aureus* by Normal Human Epidermal Keratinocytes (NHEK). **(A)** Representative Amnis^®^ Image Stream Analysis of NHEK co-cultured with 10^7^ CFU/ml ^FITC^LiSA for 1 h at 37°C, then treated for 30 min with 2% penicillin/streptomycin (P/S) to eliminate extracellular bacteria and cultured for a further 4 h prior to staining and analysis. Red stain: Alexa Fluor^®^ 647 mouse anti-human cytokeratin 14/15/16/19. Green stain: ^FITC^*S. aureus.* **(B)** Representative confocal microscopy 2D image of NHEK after co-culture with 10^7^ CFU/ml ^GFP^*S. aureus* for 1 h at 37°C. **(C)** Representative immunofluorescent staining of healthy human skin explant after 3 h infection with ^GFP^*S. aureus* (10^7^ CFU/ml) at 37°C. Red: anti-rabbit claudin-1 antibody. Green: ^GFP^*S. aureus.* Yellow box (left panel) highlights area of right panel magnified. All data are representative of three independent experiments. Images taken at 40× magnification. Scale bars represent 100 μm.

Secondly, we consolidated these findings using inherently fluorescent GFP-expressing *S. aureus* by demonstrating that co-localization of *S. aureus* and NHEK observed by flow cytometry was due to internalization, using both Amnis^®^ imaging and confocal microscopy (Figures 1B, 2 and Supplementary Video S1). Confocal micrographs clearly show the presence of bacteria not only at the cell surface, but also in the process being internalized (white arrow, Figure 1B) and deep within the cytoplasm of human keratinocytes (blue arrow, Figure 1B and Supplementary Video S1).

Lastly, to confirm that internalization by NHEK is not unique to a particular *S. aureus* strain, we demonstrated that there were no significant differences in internalization by NHEK between two clinical strains (Clin1-SA and Clin2SA: NCTC2669) and two laboratory strains (Lab1-SA: SH1000 and Lab2 SA:GFP-SA) (Figure 1Ci) (*P* < 0.0001). Furthermore, there was no detectable internalization of SE by NHEK. These data strongly support the premise that human skin keratinocytes internalize *S. aureus* but not SE, and that internalization is not specific to any one *S. aureus* strain.

To confirm that internalization was an active process involving the fibronectin binding protein (FnBP) – integrin α5β1 pathway we demonstrated that internalization of *S. aureus* FnBP mutant is reduced by over 50% (*P* < 0.0001). Furthermore, co-incubation of *S. aureus* with a FnBP-α5β1 integrin neutralizing antibody reduced internalization by 80% (*P* < 0.0001), as previously shown in HaCaT cells (Classen et al., 2011; Figure 1Cii).

For *S. aureus* to be internalized *in vivo* by keratinocytes of the deeper epidermis, the bacteria must be able to pass through the superficial stratum corneum. To demonstrate the clinical relevance of our findings, we confirmed previous studies (Mempel et al., 2002; Sayedyahossein et al., 2015; Josse et al., 2017) that ^GFP^*S. aureus* can be found deep within the epidermis of intact human skin organ culture (Figure 2C).

### Internalization of *S. aureus* by NHEK Does Not Induce Cytotoxicity in Host Cells or Release of the IL-33 Danger Signal

**FIGURE 3:**
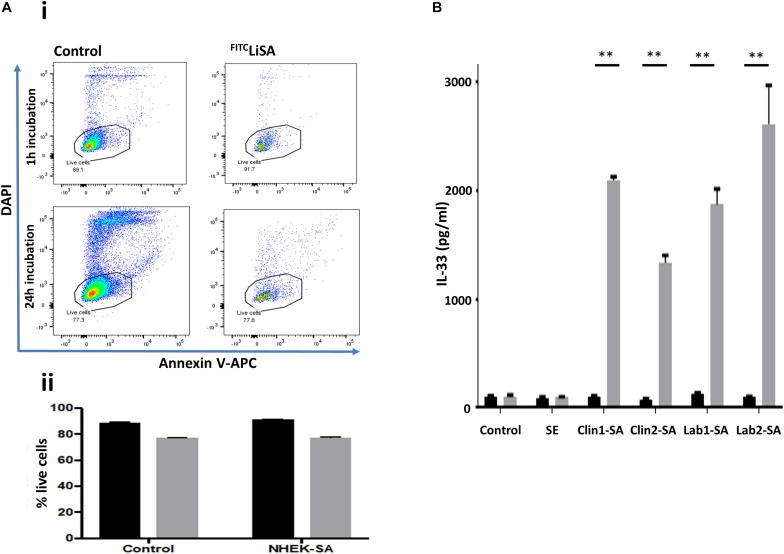
Internalization of *S. aureus* into human keratinocytes does not induce cytotoxicity, or IL-33 release by the host cells. **(Ai)** NHEK incubated with *S. aureus* for 1 h before removing extra-cellular bacteria did not lead to increased apoptosis (Annexin V) or necrosis (DAPI) staining. Representative flow cytometry plots showing Control: keratinocytes incubated in media alone, and keratinocytes incubated with *S. aureus* for 1 h, treated with penicillin/streptomycin and then incubated in media alone for a further 24 h. Gate shows DAPI^–^/Annexin V^–^ cells. **(Aii)** Bar graphs collating data from three independent experiments each done in triplicate. Black bars: 1 h, gray bars: 24 h incubation of internalized *S. aureus* and NHEK. There was no significant difference between control and NHEK-*S. aureus* (SA) groups. **(B)** IL-33 release induced in NHEK by extracellular, but not intracellular clinical and laboratory *S. aureus* isolates. Gray bars: 24 h co-culture of *S. aureus* and NHEK. Black bars: 1 h co-culture followed by P/S treatment. Twenty-four supernatants were collected and analyzed by ELISA. Data are representative of three independent experiments each performed in triplicate. Mean ± standard error of the mean. ^∗∗^*P* < 0.001, *P*-values were determined by two-way ANOVA with Dunnett’s or Tukey’s *post hoc* test.

The downstream effects of internalized *S. aureus* are poorly understood. Published data suggest that invasion of keratinocytes by *S. aureus* can induce cytotoxicity and may drive inflammatory responses, suggesting that internalization is part of the pathogenic repertoire of *S. aureus* (Mempel et al., 2002). Contrary to these previous findings, we demonstrated that *S. aureus* is not only internalized by NHEK, but that internalization does not trigger a noticeable cytotoxic effect over the subsequent 24 h, as measured by Annexin V and DAPI staining; FITC positive keratinocytes are neither Annexin V or DAPI positive (Figure 3A). Furthermore, while extracellular *S. aureus* induce large amounts of IL-33 release (Figure 3B, gray bars), internalized *S. aureus* do not induce release of this alarmin (Figure 3B, black bars). SE was not internalized and did not induce release of IL-33 by NHEK.

### Sensitivity of Internalized *S. aureus* to Common Staphylococcal Antibiotics

**FIGURE 4:**
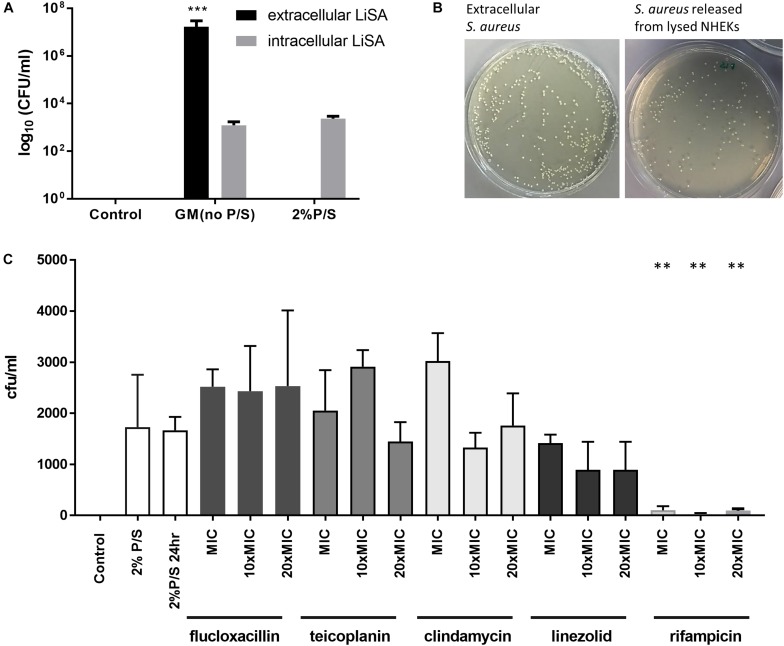
Antibiotic sensitivity of *S. aureus* internalized by human keratinocytes. **(A)** NHEK were co-cultured with 10^7^ CFU/ml Clin1-SA for 1 h at 37°C. Cells were then washed and treated with 2% penicillin/streptomycin (P/S) for 1 h to eliminate extracellular bacteria, before further culture for 24 h. The number of CFU of bacteria from supernatant (black bars) or lysed NHEK (gray bars) was quantified on nutrient agar. **(B)** Representative *S. aureus* growth on nutrient agar plate from supernatant (left panel) and lysed NHEK (right panel). **(C)** Bactericidal effects of anti-staphylococcal antibiotics (flucloxacillin, teicoplanin, clindamycin, linezolid, and rifampicin) on internalized *S. aureus* after 24 h of antibiotic treatment. Data are representative of three independent experiments performed in triplicate. Mean ± standard error of the mean. *P-*values were determined by one-way analysis of variance with Dunnett’s *post hoc* test. ^∗∗^*P* < 0.001 compared with 2% P/S at 24 h.

The heart of our study was to evaluate the ability of routinely available anti-staphylococcal antibiotics to eradicate methicillin-sensitive *S. aureus*, which had been taken up by skin keratinocytes. After allowing *S. aureus* to be taken up by NHEK for 1 h and then eradicating remaining extracellular bacteria by incubating the culture for a further hour with P/S, lysis of the keratinocytes after a further 24 h culture resulted in release of viable bacteria which could be quantified in agar culture (Figure 4A). We confirmed that the cell supernatant prior to lysis was sterile if preincubated with 2% P/S for 1 h (Figure 4A). After lysing NHEK, viable *S. aureus* were released and could be cultured. The morphology of the *S. aureus* released from the lysed NHEK was similar to those of non-internalized bacteria (Figure 4B).

We then evaluated the ability of five anti-staphylococcal antibiotics of different classes [β-lactam P (flucloxacillin), semisynthetic glycopeptide (teicoplanin), semisynthetic lincosamide (clindamycin), oxazolidinone (linezolid), and an ansamycin (rifampicin)] routinely used in clinical practice, to kill intracellular *S. aureus*. All five staphylococcal antibiotics tested were bactericidal for extracellular methicillin-sensitive *S. aureus* in concentrations ranging from 0.1 to 2 mg/ml (Table 1). However, flucloxacillin, teicoplanin, clindamycin, and linezolid had little effect on the growth of *S. aureus* which had been internalized even at 20 times the MIC (Figure 4C). In contrast, rifampicin almost completely inhibited the growth of internalized bacteria at the MIC of non-internalized bacteria (Figure 4C).

**TABLE 1:** Comparative killing of extracellular and intracellular *S. aureus* by anti-staphylococcal antibiotics.

**Antibiotic**	**Bacterial killing**	
	
	**MIC (mg/ml)^a^**	**MIC (mg/ml)^b^**
Flucloxacillin	0.12	>20-fold higher
Clindamycin	0.25	>20-fold higher
Linezolid	4.00	>20-fold higher
Teicoplanin	4.00	>20-fold higher
Rifampicin	0.5	MIC^c^

*Minimum inhibitory concentration (MIC) of antibiotics that prevented visible growth of bacteria. ^*a*^Microtitre broth dilatation method. ^*b*^Miles and Misra method. ^*c*^Rifampicin reduced bacterial viability by 99.9% compared to penicillin/streptomycin.*

## Discussion

Although the concept of *S. aureus* internalization is not new (Mempel et al., 2002; Sayedyahossein et al., 2015; Nakatsuji et al., 2016) and internalization has previously been suggested to act as a nidus for recurrent infections (Kubica et al., 2008; Marbach et al., 2019), the key conceptual advance of our study is to show that most anti-staphylococcal antibiotics used clinically are unable to effectively kill these bacteria once they have been internalized by keratinocytes. The exception is rifampicin. We deliberately chose to use methicillin-sensitive and avoid methicillin-resistant *S. aureus* in our experiments to allow for comparison of a wide range of antibiotics including flucloxacillin, which would otherwise have been excluded by the inherent bacterial antibiotic resistance of MRSA.

We also clearly demonstrate that internalization is not a ubiquitous feature of all skin staphylococci, as although both clinical and laboratory *S. aureus* strains were taken up by primary skin keratinocytes via a FnBP-α5β1 integrin dependent pathway, the skin commensal SE, which does not express FNBP, was not internalized (Sinha et al., 2000). This is in contrast to previous studies, which suggest that SE can be taken up by HaCaT transformed, aneuploid, immortalized keratinocytes (N’Diaye et al., 2016). Furthermore, in contrast to previous studies, which suggest that *S. aureus* may induce cytokine responses once internalized by HaCaT keratinocytes (Strobel et al., 2016), we demonstrated that *S. aureus* internalized into primary human keratinocytes induced neither cytotoxicity nor IL-33 release. Thus, our data suggest that HaCaT keratinocytes may not be a reliable model to study microbe–host interaction in the skin. Overall, our study highlights how internalization provides a sanctuary for *S. aureus* within skin cells, where they coexist in symbiosis with the skin keratinocytes, while at the same time avoiding killing by antibiotics.

The concept of metabolically dormant and semi-dormant bacilli residing in host intracellular niches is not new. *Neisseria meningitidis* is known to be internalized by human airway epithelial cells through a mechanism also partly dependent on actin polymerization (Toussi et al., 2016). *Mycobacteria tuberculosis* survive and grow within macrophages, which provide a sanctuary protecting these bacteria from the bactericidal effects of some anti-tuberculous antibiotics (Jindani et al., 2003). Similarly, although MIC predicts direct anti-bactericidal properties of antibiotics important for the initial cull of extracellular *M. tuberculosis*, MIC is less useful in determining antibiotic bactericidal potential against internalized bacteria.

Rifampicin is recommended for eradication of meningococcal carriage and for targeting intracellular mycobacteria (Deal and Sanders, 1969). Despite its efficacy, solitary use of rifampicin rapidly leads to antibiotic resistance (Riedel et al., 2008; Thwaites et al., 2018). Thus, its sole use in treating bacterial infections should be avoided. For tuberculosis, rifampicin is used in combination with other antibiotics, and a similar strategy might be considered for patients with recurrent staphylococcal skin infections. The half-life of rifampicin is only 3 h. Further studies are required to determine if it is the high peak concentration or the duration of treatment that is most important in eradicating the intracellular bacteria (Gumbo et al., 2007).

The fact that even 20-fold higher concentrations of some anti-staphylococcal antibiotics are ineffective in killing internalized bacteria is an important message for clinicians treating patients with these infections. The standard MIC assay is a poor determinant of antibiotic sensitivity against these internalized microbes, the exception being rifampicin, which has similar MIC for both extracellular and internalized bacteria. The most likely explanation for the effective killing of non-internalized *S. aureus* but not internalized *S. aureus* is an antibiotic’s inability to penetrate the NHEK cell membrane in high enough concentration. This possibility has previously been suggested by the variable penetration of antibiotics into neutrophils (Sandberg et al., 2010; Bongers et al., 2019). Nanoparticles might provide a delivery system for more effective eradication of intracellular *S. aureus* (Yang et al., 2018).

Current anti-microbial strategy for skin infections involves a three-pronged approach: (1) meticulous antisepsis and hygiene, (2) antibiotic stewardship, and (3) novel drug development (Cox and Worthington, 2017). Our study highlights a fourth therapeutic approach in the control and eradication of *S. aureus* causing acute skin infections. Re-colonization of the skin by *S. aureus* may not only be driven by cross-infection, but also by release of viable intracellular bacteria from keratinocytes. *S. aureus* should be considered one of the microbes that behave as a *Ghost in the Machine.* Effective killing of both intracellular and extracellular microbes are likely to be important considerations in the prevention of recurrent infections and the development of antibiotic resistance.

## Data Availability Statement

The datasets analyzed in this manuscript are not publicly available. Requests to access the datasets should be directed to PA, peter.arkwright@manchester.ac.uk.

## Author Contributions

PA, JP, CO’N, and AAK conceived and designed the study. AAK, MN, AA, and CE-C carried out the experimental work. All authors contributed to drafting and refining the manuscript.

## Conflict of Interest

The authors declare that the research was conducted in the absence of any commercial or financial relationships that could be construed as a potential conflict of interest.

## Supplementary Material

VIDEO S1 Three-dimensional rotating video image of NHEK with intracellular *S. aureus* (pink) and extracellular *S. aureus* (blue). Aerial view showing cross section of NHEK cells containing GFP-*S. aureus*. Image artificially colored. Animation created using Imaris X64 9.2.1 (Bitplane, Oxford, United Kingdom).
